# Evaluation of AI-Powered Routine Screening of Clinically Acquired cMRIs for Incidental Intracranial Aneurysms

**DOI:** 10.3390/diagnostics15030254

**Published:** 2025-01-22

**Authors:** Christina Carina Schmidt, Robert Stahl, Franziska Mueller, Thomas David Fischer, Robert Forbrig, Christian Brem, Hakan Isik, Klaus Seelos, Niklas Thon, Sophia Stoecklein, Thomas Liebig, Johannes Rueckel

**Affiliations:** 1 Institute of Neuroradiology, University Hospital, LMU Munich, 81377 Munich, Germany; christina.schmidt@med.uni-muenchen.de (C.C.S.);; 2 Department of Radiology, University Hospital, LMU Munich, 81377 Munich, Germany; 3 Department of Neurosurgery, University Hospital, LMU Munich, 81377 Munich, Germany

**Keywords:** artificial intelligence, intracranial aneurysms, MRI, screening, TOF-MRA

## Abstract

**Objectives**: To quantify the clinical value of integrating a commercially available artificial intelligence (AI) algorithm for intracranial aneurysm detection in a screening setting that utilizes cranial magnetic resonance imaging (cMRI) scans acquired primarily for other clinical purposes. **Methods**: A total of 907 consecutive cMRI datasets, including time-of-flight-angiography (TOF-MRA), were retrospectively identified from patients unaware of intracranial aneurysms. cMRIs were analyzed by a commercial AI algorithm and reassessed by consultant-level neuroradiologists, who provided confidence scores and workup recommendations for suspicious findings. Patients with newly identified findings (relative to initial cMRI reports) were contacted for on-site consultations, including cMRI follow-up or catheter angiography. The number needed to screen (NNS) was defined as the cMRI quantity that must undergo AI screening to achieve various clinical endpoints. **Results**: The algorithm demonstrates high sensitivities (100% for findings >4 mm in diameter), a 17.8% MRA alert rate and positive predictive values of 11.5–43.8% (depending on whether inconclusive findings are considered or not). Initial cMRI reports missed 50 out of 59 suspicious findings, including 13 certain intradural aneurysms. The NNS for additionally identifying highly suspicious and therapeutically relevant (unruptured intracranial aneurysm treatment scores balanced or in favor of treatment) findings was 152. The NNS for recommending additional follow-/workup imaging (cMRI or catheter angiography) was 26, suggesting an additional up to 4% increase in imaging procedures resulting from a preceding AI screening. **Conclusions**: AI-powered routine screening of cMRIs clearly lowers the high risk of incidental aneurysm non-reporting but results in a substantial burden of additional imaging follow-up for minor or inconclusive findings.

## 1. Introduction

The potential of artificial intelligence (AI)-based medical image analysis to mimic or partially overcome medical experts’ diagnostic performance has been demonstrated in various scenarios [1,2,3,4,5]. Nevertheless, the successful translation of AI to daily routines necessitates a strategic deployment to suitable clinical scenarios [6,7,8,9]. One promising AI deployment scenario might be the analysis of high-volume and standardized medical imaging for incidental findings that are prone to be missed because they commonly occur outside the assessing radiologists’ primary focus [10,11].

Screening cross-sectional imaging for incidental intracranial aneurysms might be a promising AI application for the following key factors: Firstly, there is a notable disease prevalence estimated to range between 1% and 7% [12,13,14,15]. Secondly, there is clinical relevance; despite often lacking initial symptoms, intradural aneurysms carry a risk of rupture, leading to subarachnoid hemorrhage (SAH) with high fatality rates [up to 66.7% [15,16,17]] and long-term morbidity implications [18,19,20]. For that reason, the early detection of incidental aneurysms is crucial to prevent SAH-related mortality and morbidity [21,22], as endovascular and surgical therapies are available [23]. Lastly, the increasing volume of cranial magnetic resonance imaging (cMRI) scans for various clinical purposes raises the chance of early aneurysm detection [11,24]. However, studies indicate that even experienced radiologists frequently fail to report incidental aneurysms, especially those of smaller sizes [24,25].

Recent studies have demonstrated high-performance AI algorithms for the detection of intracranial aneurysms in cross-sectional imaging, such as (time of flight [TOF]) magnetic resonance imaging [26,27,28,29,30,31,32,33] or computed tomography angiography (CTA) [34,35,36]. However, many of these validation studies have methodological limitations, such as small sample sizes [26,30], reference standards of limited quality [26,29] or the utilization of pathology-enriched validation cohorts that often do not sufficiently consider small aneurysm sizes [30] and especially do not reflect clinical routine prevalence [27,28,31,32]. To our knowledge, only one study investigated the delivery of an aneurysm-detecting algorithm into a real-world setting; however, it was limited to CTA analysis and the study utilized an algorithm that is not commercially available [34].

This study aims to overcome many of the limitations mentioned above and extends beyond basic algorithm characterization. To our knowledge, it represents the first validation study that aims to quantify the added clinical value of a commercially available AI algorithm in a clinically representative screening scenario based on cMRI scans that are anyway acquired for various clincial reasons. In this second reader-screening scenario, two primary endpoints were established: (A) the number of screening cMRIs required to identify an additional clinically relevant aneurysm, and (B) the number of screening cMRIs needed to detect findings of varying relevance that still necessitate subsequent imaging follow-up or further evaluation. To achieve this, a cohort of retrospectively identified cMRIs (including TOF-MRA) of consecutive patients was established and a high-quality reference standard was derived from consultant-level neuroradiologists’ re-assessments, including aneurysm-specific workup recommendations. Additionally, the clinical relevance of AI-detected findings was evaluated by comparing them with the initial cMRI reports, and with respect to newly detected findings and the prospective part of our study, by reaching out to patients and offering an individual consultation and risk stratification including further imaging such as another cMRI or an additional catheter angiography. In doing so, this study finally quantifies the number of cMRIs that need to be screened (NNS) by an AI algorithm to additionally detect clinically relevant findings: We anticipate a high NNS to additionally detect aneurysms with an indication for treatment and a much lower NNS to additionally detect small or inconclusive findings that nevertheless necessitate further imaging follow-up. Regarding the resulting effects of deploying an AI-based routine screening of cMRIs acquired for other clinical reasons, this methodology allows us to balance the benefit of preventively treating or following up a few additionally detected aneurysms against a possibly high burden of additionally indicated imaging follow-ups for inconclusive or small findings.

## 2. Materials and Methods

Approval of the institutional ethics committee was obtained for this study (approval number 23-0342).

### 2.1. Study Cohort and Data Acquisition

**Figure 1:**
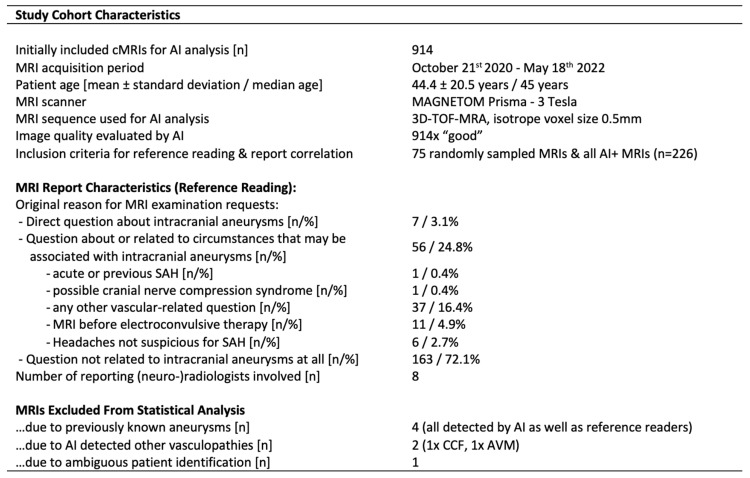
**Study Cohort Characteristics.** 914 consecutive cMRIs (all acquired at the same scanner including the same TOF-MRA sequence parameters as specified below) were included for AI analysis. 226 MRIs were included in the reference reading and report correlation, thereby 75 randomly sampled cMRIs as well as all cMRIs with at least one AI detection suspicious for an intracranial aneurysm (see Figure 2a). Seven cMRIs were excluded from the following statistics due to other AI-detected vascular malformations (1 nidal arteriovenous malformation, 1 possible carotid-cavernous-fistula), due to previously known intracranial aneurysms (4 aneurysms of 4 different patients, all of which were detected by AI as well as by the reference readers) or for technical reasons (1 ambiguous retrospective patient/report identification). Patients and cMRI report characteristics as illustrated above. Abbreviations: AI—artificial intelligence; AI+—MRI with AI detections; CCF—carotid-cavernous-fistula; AVM—arteriovenous malformation; SAH—subarachnoid hemorrhage.

All patients who underwent cMRI imaging from 10/2020 to 05/2022 with our standard cMRI protocol (including a high-resolution TOF-MRA) at a specific 3 Tesla MRI scanner (MAGNETOM PRISMA, Siemens Healthineers, Forchheim, Germany) were consecutively included. The MRI scanner is exclusively dedicated to patients in the psychiatric department of our major university hospital. In cases where multiple cMRIs were performed on the same patient, only the initial cMRI was included. No other exclusion criteria have been initially applied. A total of 914 consecutive cMRI datasets of 914 different patients were included (see Figure 1). Our data inclusion strategy aimed to deliberately target incidental findings in high-quality cMRIs, all initially assessed by neuroradiology experts, without any focus on aneurysm- or vascular-related high-risk patient subgroups, as the primary purpose of the enrolled cMRI scans was to rule out organic causes of psychiatric disorders.

The cMRI datasets were pseudonymized, exported in DICOM format, and analyzed by an AI algorithm designed for aneurysm detection in TOF-MRAs (algorithm description see below). Pseudonymized cMRI datasets, along with the illustrated AI results, were reintegrated into our Picture Archiving and Communication System (PACS) for the reference reading.

**Figure 2:**
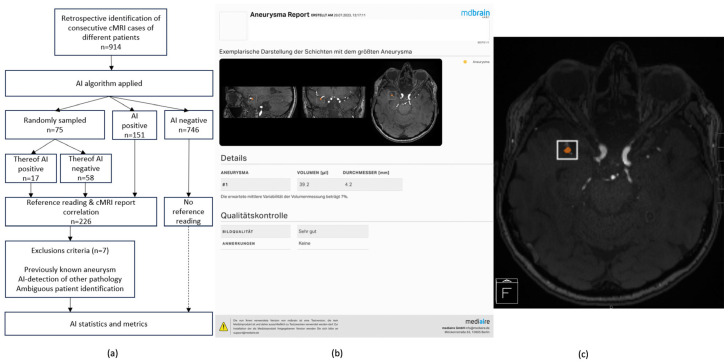
**Study Cohort Enrollment Flowchart and AI-Generated Result Illustration.** (**a**): Enrollment flowchart illustrating AI analysis and reference readers’ re-assessments of 914 initially included consecutive cMRIs (914 different patients). (**b**): Exemplary illustration of an AI report as provided for each analyzed cMRI. The largest detection is shown in the three orthograde planes. Volumes and diameters are shown for all detections based on AI segmentation. (**c**): AI detections are additionally illustrated by bounding boxes in TOF-MRA duplicates. Abbreviations: AI—artificial intelligence; AI Positive—cMRIs with AI detections; AI Negative—cMRIs without AI detections; cMRI—cranial magnetic resonance imaging; TOF-MRA—time-of-flight magnetic resonance angiography.

Seventy-five cMRIs selected by simple randomization (17 AI-positive and 58 AI-negative), in addition to all remaining cMRIs with at least one AI detection (*n* = 151), underwent the blinded reference reading. Pseudonymized cMRIs were distributed among three consultant-level neuroradiologists (>11/10/5 years of experience in diagnostic and interventional neuroradiology) who were blinded to patient identifying data (including patient age, therefore assuming a significantly remaining life expectancy) as well as blinded to the initial cMRI reports. Reference readers were neither involved in the study design nor in the study cohort establishment, had full access to all initially obtained cMRI sequences and were additionally provided with the AI results illustrated as secondary captures in TOF-MRA duplicates as well as presented as tabular data containing segmentation volumes and diameters of detected findings (see Figure 2b,c). Reference readers assessed the datasets regarding intracranial aneurysms, including a plausibility check of AI detections. For this purpose, Likert-based confidence scores were used for all suspicious and/or AI-detected findings as follows: 0—no aneurysm, 1—aneurysm unlikely, 2—aneurysm likely, and 3—certain aneurysm. Those AI detections that were not confirmed by the reference readers (score 0) were further categorized based on whether localized at an intracranial artery or not. Findings suspicious for an intracranial aneurysm (scores 1–3) were categorized according to localization (reference reading localization categories: ICA extradural, ICA infraclinoidal, ICA paraophthalmic, ICA supraophthalmic, Pcom origin, AchA origin, ICA bifurcation [“carotid T”/terminal ICA), MCA, ACA, Acom, other localization in the anterior circulation, V4 segment, PICA territory, basilar artery, basilar tip, SUCA origin, PCA territory, other localization in the posterior circulation], morphology (saccular/fusiform/dysplastic-mixed), size (AI-based volumetry or manual maximal diameter measurement of those findings not detected by the AI) and aneurysm thrombosis (yes/no). Finally, reference readers recommended a further workup strategy individually for each suspicious finding (“no consequence”/“non-invasive follow-up imaging, e.g., MRI”/“further evaluation by catheter angiography”).

The initial cMRI reports were reviewed by well-trained medical students and categorized according to underlying imaging indications, involved reporting (neuro-)radiologists and reported findings suspicious for intracranial aneurysms.

Basic cohort characteristics are elucidated in Figure 1. The study design and enrollment flow-chart as well as the way how AI results have been illustrated for the reference readers are illustrated in Figure 2.

### 2.2. Patient Consultation in Case of Additionally Detected Findings

Patients diagnosed with newly detected findings through this study (based on AI analysis, the dedicated reference reading and the initial cMRI report evaluation) have been reached out to and offered a neuroradiological on-site consultation. Individual patient consultations were based on rupture risk assessment [ISUIA [22]/UCAS [37] /PHASES [38] scoring] as balanced to treatment risks [UIATS recommendations [39]] considering both patient-specific medical conditions and aneurysm characteristics (as far as possible based on cMRI imaging). Individual workup strategies were established jointly with the patients. They included new cMRI follow-ups and/or an additional catheter angiography as well as interdisciplinary case discussions for follow-up and/or therapy recommendations. When patients could not be reached after multiple attempts, an aneurysm risk stratification (UIAT scoring system) was roughly estimated based on cMRI-determined aneurysm location and size and patient-specific risk factors as far as accessible in the existing medical records.

### 2.3. Artificial Intelligence Algorithm

The imaging studies were analyzed by the artificial intelligence-based software mdbrain version 4.8 (Mediaire GmbH, Berlin, Germany), a commercially available medical product designed to assist radiologists in reading cMRI datasets. The underlying aneurysm segmentation algorithm is based on a 3D convolutional neural network with a U-NET architecture [40]. The model was trained on >400 cMRI datasets of both healthy subjects and subjects with unruptured saccular cerebral aneurysms. For each subject, the data consisted of a TOF-MRA scan as well as a corresponding binary mask of the aneurysms, as segmented by an expert radiologist. Before training, all datasets were resampled to a fixed spacing before the intensity was normalized per image for zero mean and unit variance. During training of the neural network (using stochastic gradient descent), the input was provided in batches of patches, in which it was ensured that some patches contained aneurysm voxels and others did not. Augmentation was performed spontaneously during training on the input patches to increase the generalization ability of the neural network. The training data set did not contain images of the institutions that externally validated the algorithm in this study.

An algorithm analysis report based on a binary classification (no confidence assessment of suspected findings) is illustrated in Figure 2, including representative images and quantitative measurements (volume, diameter).

### 2.4. Statistical Workup

The statistical analysis was focused on newly detected suspicious findings that were not previously identified. The primary objectives were to quantify the number of screening cMRIs necessary to detect one additional clinically significant finding or to identify a finding that justifies further imaging follow-up/workup. Seven cMRIs were excluded from statistical evaluation for the reasons outlined in the results section and illustrated in Figure 1.

The AI algorithm performance compared to the neuroradiologists’ reference reading was assessed using descriptive statistics and common diagnostic metrics such as sensitivity and predictive values. Subgroup analyses were conducted based on aneurysm size and localization. Binary reference standards (RFS) have been built for statistical purposes by pooling the reference reading confidence scores (0–3) as follows: The most specific RFS I summarizes the confidence scores 0–2 as negative for an intracranial aneurysm, therefore focusing solely on certain aneurysms (score 3). Conversely, the most sensitive RFS III classifies the confidence scores 1–3 as positive for intracranial aneurysms, only neglecting the completely non-suspicious findings. The intermediate RFS II was constructed accordingly, classifying scores 2 and 3 as positive.

The added clinical value of a possible AI-powered routine screening was evaluated by comparing the findings reasonably detected by the AI with those already described in the original cMRI reports. Common descriptive statistics were utilized for quantification. In this context, the *number needed to screen* (NNS) was defined as the number of study cohort cMRIs that must undergo AI screening to uncover at least one additional observation that was not described by the initial cMRI report. The NNS was calculated for findings of differentially defined clinical significances, e.g., related to aspects of the reference readers’ assessments or related to UIATS-based aneurysm risk stratifications.

## 3. Results

### 3.1. Cohort Characteristics

The patient age was 44.4 ± 20.5 years (mean ± standard deviation) with a median age of 45 years. The TOF-MRA image quality as assessed by the AI algorithm was good in all 914 initially enrolled cMRIs. The original reasons for cMRI requests were reviewed for all 226 patients who qualified for the reference reading according to the criteria as outlined in the methodology section. Over 96% of these cMRIs were initially requested without any direct question about the presence of intracranial aneurysms, as elucidated in detail in Figure 1.

Seven patients have been excluded from further statistical analysis due to other (AI-detected) vascular malformations/pathologies (*n* = 2), due to aneurysms that were already known before the initial cMRI was acquired (*n* = 4) or for technical reasons (*n* = 1), as shown in Figure 1. Statistical analysis was based on the remaining cohort of 907 cMRI datasets.

### 3.2. AI Algorithm Performance

**Figure 3:**
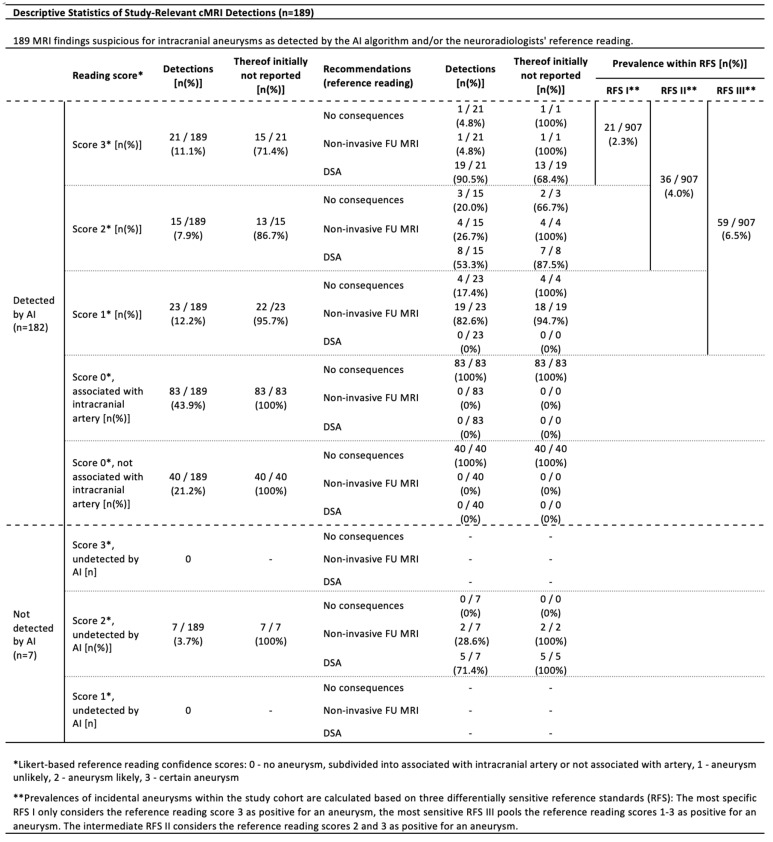
**Descriptive Statistics of Study-Relevant cMRI Detections.** Statistics of 189 MRI findings suggestive of incidental intracranial aneurysm (detected by AI and/or reference readers) across 219 different MRIs/patients. The findings have been extracted from a total of 907 cMRI datasets that have been screened by the AI algorithm and a neuroradiologists’ reference reading of all AI+ cMRI datasets, in addition to an initial sample of 75 cMRI datasets (see Figure 2a). Reference readers categorized suspicious cMRI findings utilizing the above-mentioned Likert-based confidence scoring *. Resulting consequence recommendations are categorized into “No consequences”, “Non-invasive FU MRI” and “DSA in terms of a possible therapy relevance”. All possible aneurysms without any workup recommendation refer to extradural paracavernosal localizations. Abbreviations: AI—artificial intelligence; AI+—cMRIs with AI detections; cMRI—cranial magnetic resonance imaging; FU MRI—follow-up magnetic resonance imaging; DSA—digital subtraction angiography; RFS—reference standard.

**Figure 4:**
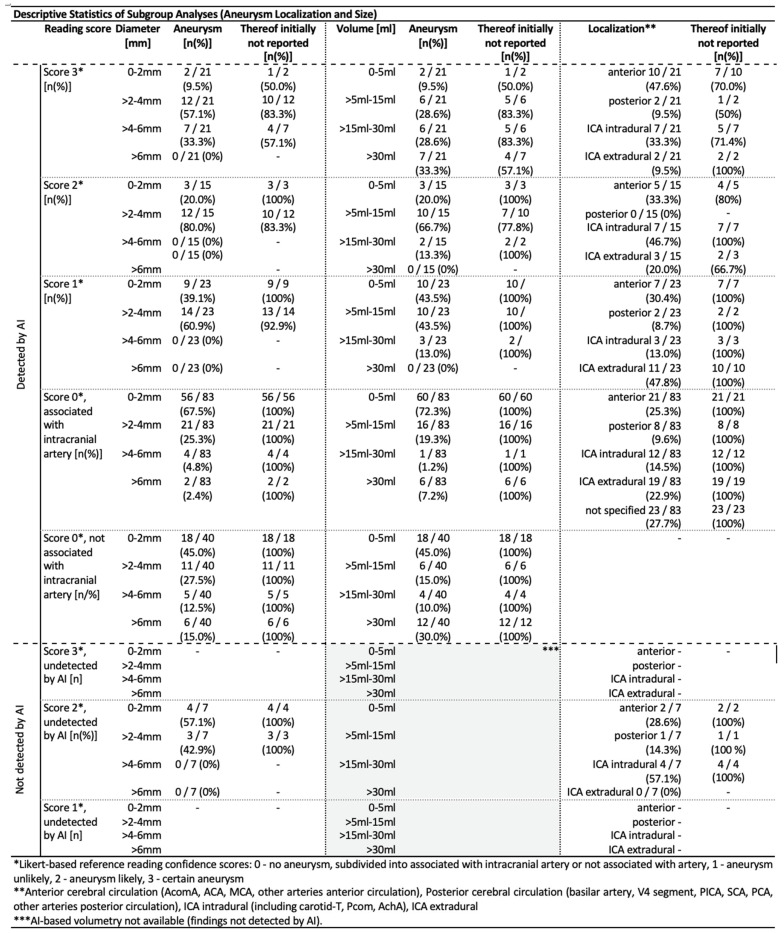
**Descriptive Statistics of Subgroup Analyses (Aneurysm Localization and Size).** Illustration scheme similar to Figure 3 with subgroup analysis according to aneurysm size (diameter and volume according to AI-based segmentations) and localization according to the reference readers’ assessments. Aneurysm localizations were grouped into categories as mentioned above **. Abbreviations: AI—artificial intelligence; ICA—internal carotid artery; Acom—anterior communicating artery; ACA—anterior cerebral artery; MCA—middle cerebral artery; PICA—posterior inferior cerebellar artery; SCA—superior cerebellar artery; PCA—posterior cerebral artery; Pcom—Posterior communicating artery; AchA—anterior choroidal artery.

In the resulting cohort of 907 statistically analyzed cMRIs, the AI algorithm detected 182 findings (0.4 mm to 10.6 mm in diameter) across 161 different MRAs, representing an algorithm alert rate of 17.8% (161/907). This overcomes the estimated prevalence of aneurysms, ranging from 2.3% (21/907) to 6.5% [(21 + 15 + 23)/907] within our study cohort based on the reference reading and depending on whether inconclusive findings are considered or not (see Figure 3). The majority of 67.6% [123 (83 + 40) out of 182] of AI detections was deemed non-relevant (score 0) by the reference readers, and the other findings (score 1–3) ranged from 0.6 mm to 5.7 mm (Figure 4). Notably, a significant subset of these findings (*n* = 40) lacked any identifiable local association with an intracranial artery (Figure 3 and Figure 4). As a result, our analysis delineates high false positive rates (FPR) ranging from 67.6% [(21 + 15 + 23)/182] to 88.5% [(182 − 21)/182], depending on the different RFSs applied.

**Figure 5:**
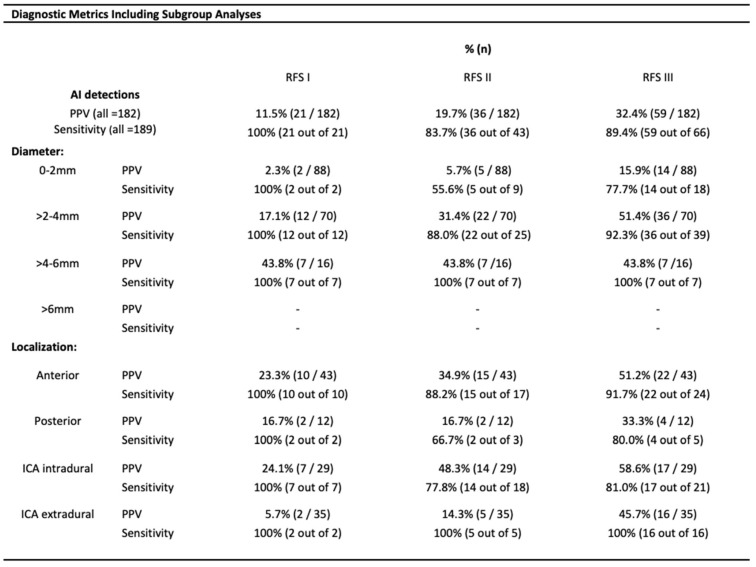
**Diagnostic Metrics Including Subgroup Analyses.** Diagnostic AI metrics have been calculated based on three differentially sensitive reference standards (RFS I-III) as already elucidated in the caption of Figure 3. PPV and sensitivity are calculated for the overall cohort as well as for different subgroups. There is a non-intuitive sensitivity drop from RFS III to the less sensitive RFS II caused by the fact that all seven findings not detected by the AI were assessed as score 2 findings by the reference readers. Abbreviations: AI—artificial intelligence; PPV—positive predictive value; RFS—reference standard; ICA—internal carotid artery.

Focusing on the limited PPVs, it was found that for 32.4% (59/182) of all AI detections, the presence of an underlying aneurysm was at least considered by the reference readers (as indicated by the scores 1–3), resulting in PPVs up to 32.4% for this most sensitive RFS interpretation (Figure 5). However, when restricting the analysis to only those findings where underlying aneurysms were considered as probable (RFS 2 + 3)/certain (RFS 3), the PPVs diminished to 19.7% [(21 + 15)/182]/11.5% (21/182) (Figure 5).

The algorithm achieved 100% sensitivity for score 3 findings of any size (certain aneurysms), but at the cost of limited PPVs, as shown in Figure 5. Including score 2 findings, 7 out of 43 suspected aneurysms (score 2/3) were missed by the AI, resulting in a sensitivity of 83.7%. All missed findings were smaller than 4 mm in diameter, identified by the reference readers but not documented in the initial cMRI reports. This finally results in an algorithm with 100% sensitivity for certain aneurysms of any size as well as for less suspicious findings larger than 4 mm in diameter.

The size of AI detections was correlated to the reference readers’ approval as follows: larger AI detections more likely drew attention to those findings that have been confirmed as suspicious for an aneurysm. For instance, more than 90% of score 3 findings were larger than 2 mm in diameter (Figure 4). In contrast, a majority of 67.5% of artery-associated score 0 findings were smaller than 2 mm in diameter (Figure 4). This ultimately results in increasing PPVs for larger AI detections, ranging from a minimum of 2.3% (for lesions smaller than 2 mm) to 43.8% (for lesions larger than 4 mm), as outlined in Figure 5.

A subgroup analysis based on aneurysm localization was performed (Figure 4 and Figure 5), but the sample sizes were too small to assess significant differences in algorithm performance by localization.

### 3.3. Clinical Benefit of an AI-Based Aneurysm Screening

The potential benefit of an AI-based routine screening for intracranial aneurysms does not only depend on the algorithm performance itself as outlined above, but also on the (neuro-)radiologists’ initial detection accuracy without AI assistance and the clinical relevance of additionally detected findings. Therefore, the initial cMRI reports were reviewed and cases with newly detected findings were correlated with workup strategies as recommended by the reference readers and/or discussed jointly with those patients that have been reached out for on-site consultation.

**Figure 6:**
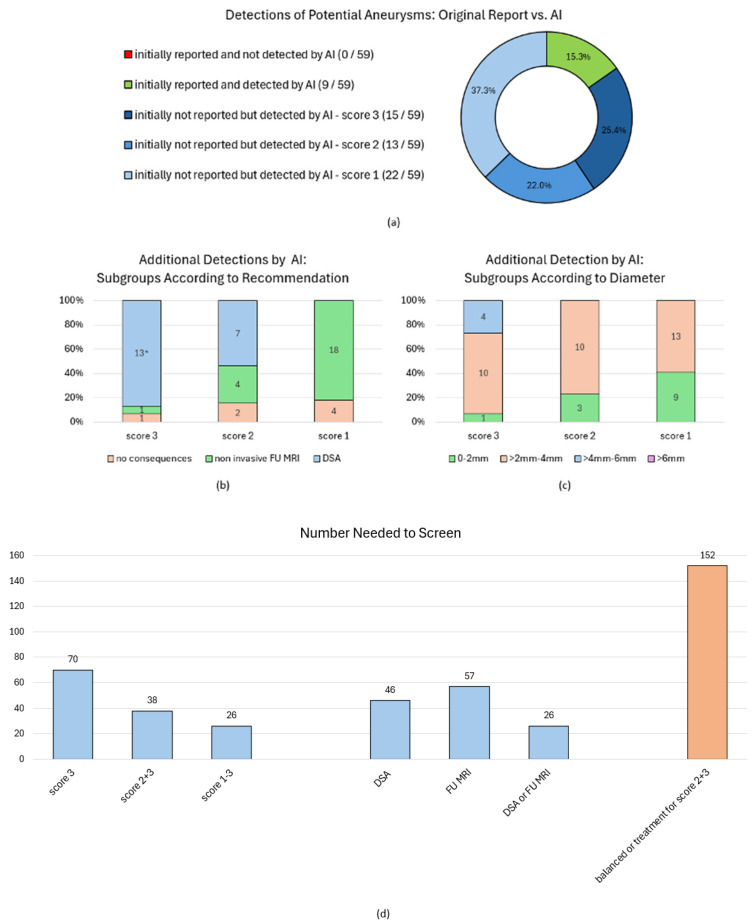
**Characteristics of Findings Additionally Detected Through This Study and Resulting Numbers Needed to Screen to Uncover These Findings of Differentially Defined Clinical Significance.** (**a**): Comparison of initially reported aneurysms and AI detections at least suspicious for an aneurysm as confirmed by the reference reading (scores 1–3). Another 123 AI detections (67.6% of all AI detections) were classified as non-suspicious. * Two out of the 15 additionally detected score 3 findings were described in the initial report, but nevertheless initially not judged to be an aneurysm (the reference readers recommended a DSA workup for both). (**b**): Workup recommendations for additionally detected findings. All suspected aneurysms without any recommended workup consequence refer to extradural paracavernosal aneurysm localizations. (**c**): Sizing categories of AI detections suspicious for intracranial aneurysms. (**d**): The number of cMRIs needed to screen (NNS) for uncovering at least one additional finding of high confidence and/or of clinical significance as outlined above. NNS calculated for considered therapeutic consequences (“balanced or treatment”) are based on UIAT score estimations as elucidated in Figure 7. Abbreviations: AI—artificial intelligence; cMRI—cranial magnetic resonance imaging; DSA—digital subtraction angiography; FU MRI—follow-up magnetic resonance imaging; NNS—number needed to screen; UIAT score—unruptured intracranial aneurysm treatment score.

The comparison of AI detections and the reference reading, in contrast with the initial cMRI reports, revealed that the algorithm accurately identified every single of these findings suspicious for an aneurysm (*n* = 9, diameters 0.7–5.7 mm) that were already described in the initial cMRI reports. Nevertheless, this refers to only 9 out of an overall 59 (15.3%) reasonable (scores 1–3) AI detections with the other initially non-reported 50 findings (84.7%) assessed by the reference readers as follows: 15 certain aneurysms (score 3, 13 intradural), 13 likely aneurysms (score 2, 11 intradural), and 22 unlikely aneurysms (score 1, 12 intradural), as shown in Figure 6a.

**Figure 7:**
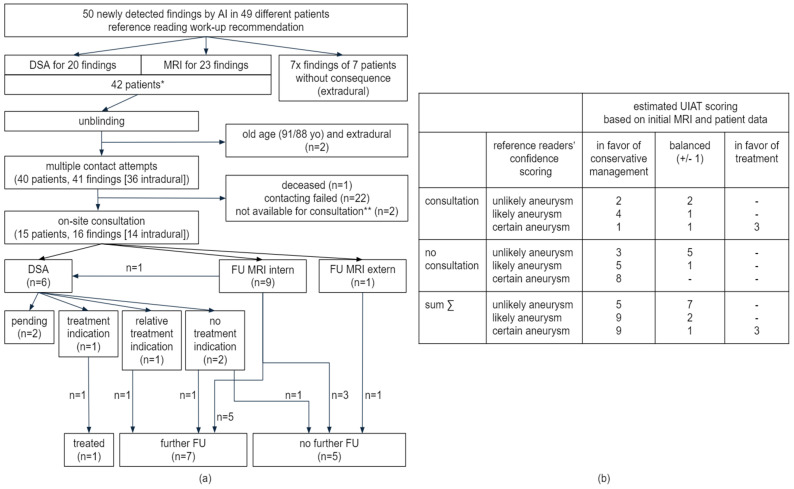
**Enrollment Flowchart for Patient On-Site Consultations and UIAT Score Estimations for Additionally Detected Findings Suspicious for Intradural Aneurysms.** (**a**): Flowchart illustration to break down the way from reference reading workup recommendations to patient on-site consultations, including imaging follow-up and finally resulting individual workup strategies. The flow chart exclusively considers those suspicious findings that have been newly detected through the AI screening of this study. * One patient with two findings with different recommendations: one DSA and one FU MRI. ** Patients were contacted by telephone but were not available for on-site consultation (FU MRI and consultation later or elsewhere was recommended). (**b**): UIAT score estimations based on MRI characteristics and on patient data and risk factors as evaluated during on-site consultations or—as far as accessible—in the existing medical records for those patients who were not available for on-site consultation. Balanced UIAT scores with differences ±1 as per definition; in favor of conservative management or treatment accordingly. Presented numbers refer to cMRI datasets (not to single findings) and are the basis for the NNS calculations as illustrated in Figure 6d. Abbreviations: AI—artificial intelligence; DSA—Digital Subtraction Angiography; FU MRI—Follow-Up Magnetic Resonance Imaging; UIAT score—unruptured intracranial aneurysm treatment score.

The majority of these newly identified findings, reasonably indicative of possible aneurysms, measured 2–4 mm in diameter, and none of these findings had a diameter exceeding 6 mm (Figure 6c). The reference readers’ workup recommendations for newly detected AI findings are illustrated in Figure 6b. In summary, the reference readers recommended a DSA workup for 20 findings and a cMRI follow-up for 23 findings, distributed across 42 different patients (Figure 7a). The remaining seven detections without workup recommendation all referred to extradural ICA localizations (Figure 6b and Figure 7a).

**Figure 8:**
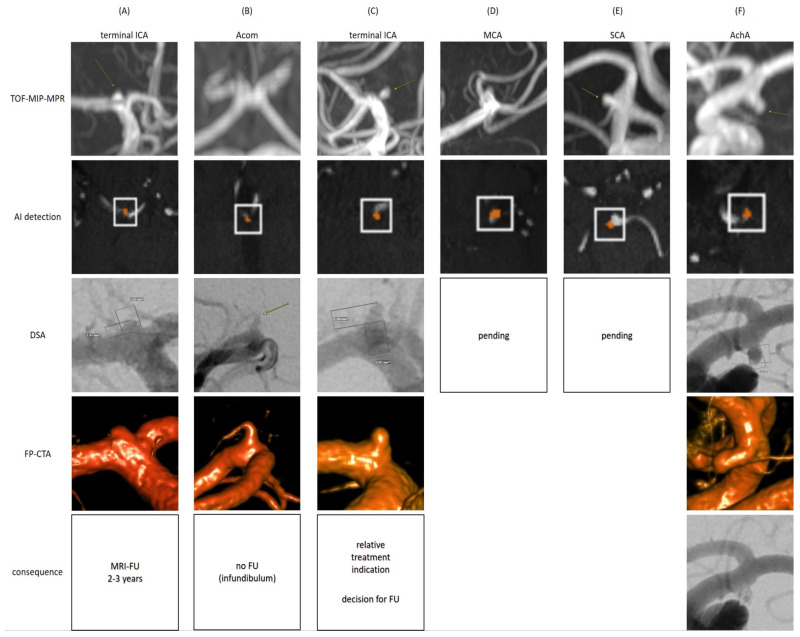
**Catheter Angiography Workups of Newly Detected Findings Suspicious for Intradural Aneurysms.** (**A**): A patient in her/his fifties with a TOF-MRA suspicious for an initial terminal ICA aneurysm who favored a catheter angiography over cMRI follow-up after consultation. The DSA demonstrated an initial aneurysm of 1–2 mm without indication for treatment. The finding will be followed up by cMRI at multi-year intervals. (**B**): A patient in her/his sixties with an inconclusive TOF-MRA finding at the Acom who favored catheter angiography over cMRI follow-up after consultation. The finding turned out to be an infundibulum without any necessity for further follow-up. (**C**): A patient in her/his thirties with a small terminal ICA aneurysm of approx. max. 2 mm as confirmed by catheter angiography. According to neurosurgical and neuroradiological consultations, there is a relative treatment indication. The patient decided to reduce risk factors by stopping smoking and will have cMRI follow-ups in a multi-year interval. (**D**): A patient in her/his thirties with a possible MCA aneurysm was scheduled for catheter angiography (pending/postponed for patient-specific reasons). (**E**): A patient in her/his sixties with a possible SUCA aneurysm was scheduled for catheter angiography (pending/postponed for patient-specific reasons). (**F**): A patient in her/his fifties with a saccular AchA aneurysm max. approx. 3.4 × 1.8 mm (dome height × neck width) as confirmed by catheter angiography. The aneurysm was treated by coil occlusion without any complications. Abbreviations: f—female; m—male; yo—years-old; ICA—internal carotid artery; Acom—anterior communicating artery; MCA—middle cerebral artery; SCA—superior cerebellar artery; AchA—anterior choroidal artery; TOF-MIP-MPR—time-of-flight maximum-intensity-projection multi-planar reconstruction; AI—artificial intelligence; DSA—digital subtraction angiography; FP-CTA—flat-panel computed tomography angiography; FU—follow-up.

All patients with newly detected findings through this study and with any workup recommendation derived from the reference reading were contacted, except for two elderly patients [91/88 years old (yo)] with very small findings. Finally, 15 out of 40 (37.5%) patients were reached out and attended an individual on-site consultation (see Figure 7a). Ten follow-up (FU) cMRIs were acquired and six catheter angiographies (DSA) were indicated (see Figure 8 with exemplary cases). Two DSA examinations are still pending for patient-specific reasons; regarding the other cases, one aneurysm was already treated, and seven findings were scheduled for further follow-up in a multi-year interval, among them one aneurysm with a relative treatment indication and the patient’s decision favoring follow-up first (Figure 7a).

The 36 additionally detected findings in intradural localization were distributed across 36 different cMRIs with resulting numbers of cMRIs needed to screen (NNS) to additionally uncover clinically relevant findings (here only focussing on intradural localizations) as follows (Figure 6d): NNSs of 70/38/26 to additional uncover one certain (score 3)/at least probable (scores 2 + 3)/any possible (scores 1–3) intradural aneurysm. The NNS for indicating any kind of recommended follow-up imaging (MRI or DSA) was 26. The NNS to find at least one additional finding with recommended DSA workup was quantified with 46. Regarding a possible therapeutic relevance of findings based on preliminary UIAT^2^ score estimation derived from cMRI characteristics and accessible patient data (on-site consultation or medical records) as illustrated in Figure 7b, the resulting NNS for detecting one “at least probable aneurysm” (scores 2 + 3) with an UIAT^2^ score balanced or in favor of treatment was 152 (Figure 6d and Figure 7b).

## 4. Discussion

The presented results quantify the clinical impact of routine cMRI screenings using a commercially available AI algorithm specifically designed for the detection of intracranial aneurysms in TOF-MRAs by leveraging cMRI scans acquired for various clinical indications. Based on our external validation, we demonstrated algorithm sensitivities of 100% for certain aneurysms of any size as well as for less suspicious findings larger than 4 mm in diameter; these metrics suggest a promising deployment into a clinical rule-out-scenario. Nevertheless, high sensitivities also result from a high MRA alert rate (17.8%), which yields limited PPVs (11.5% to 43.8%). On the one hand, a majority (50 out of 59, 84.7%) of suspicious findings, among them 13 certain aneurysms in intradural localization (NNS 70) and three of them with an estimated UIAT score in favor of treatment (NNS 303), have not been described by the initial cMRI reports. Yet, these additional relevant detections must be opposed to a high fraction of additionally detected small or inconclusive findings without any immediate therapeutic impact, that nevertheless substantially contribute to a low NNS of 26 to indicate at least one more imaging follow-/workup. This demonstrates the high burden of additional imaging that would result from a routine cMRI screening as simulated by our study.

The contextualization of our study in the scientific context is limited to the comparison of the basic algorithm performance with other validation studies. For instance, Lehnen et al. [27] externally validated an older algorithm version of the same commercial product with a lower overall sensitivity of only 72.6%, although it was based on an aneurysm-enriched cohort and without considering inconclusive findings (as represented in our study by the Likert-based confidence scoring). Other algorithms designed for intracranial aneurysm detection in TOF-MRAs have also been validated on pathology-enriched cohorts with high sensitivities ranging from 70% up to 100% [28,29,30,31,32]. The metrics comparison of non-sensitivity metrics is hampered by the fact that the usage of pathology-enriched validation cohorts by some other studies does not allow calculating prevalence-dependent metrics. In contrast, Kuwabara et al. used a series of consecutive cases and tackled the challenge of high sensitivities coupled with an unacceptably high false positive rate using an algorithmic tuning approach; however, their work remained confined to statistical performance optimization without clinical implementation/deployment [41].

In contrast to other studies, our approach extends beyond basic algorithm validation by quantifying the added value of integrating an AI-based “second reader” TOF-MRA analysis into a clinically representative aneurysm-screening scenario, with resulting clinically driven workup strategies (follow-up, treatment) for additionally detected findings as study endpoints. To our knowledge, a comparable approach has not been published to date. Hence, there is a need to discuss the methodology approach and the generalizability of our results.

In this study the NNS was used to additionally uncover relevant findings as a key measure to quantify the added clinical value of an AI-powered routine cMRI screening. However, the NNS is influenced not only by the performance of the algorithm itself but also by several other study-specific factors. Firstly, the aneurysm detection accuracy without AI support (as represented by the initial cMRI reports) affects the NNS—the lower the sensitivity of the initially reporting neuroradiologists, the lower the resulting NNS quantifications regarding additional detections. The fact that all included cMRIs were initially reported by specialized neuroradiologists might, therefore, overestimate the NNSs. Secondly, NNS quantifications are also influenced by the reference readers—a higher sensitivity in classifying AI findings as relevant will finally lower the resulting NNSs. In this context, it remains unclear whether the highly experienced neurointerventional reference readers, compared to general radiologists, discard AI findings more frequently due to lower confirmation bias or if their expertise leads to higher confirmation rates, particularly for detecting very small aneurysms. Lastly, the underlying cohort characteristics might also impact the quantified NNS. The higher the prevalence of relevant and small findings and the higher their detectability due to high image quality, the higher the risk of initial non-reporting and the lower the potentially resulting NNS quantifications. In this context, we assume an above-average image quality with only little motion artifacts in the study cohort of relatively young and predominantly cardiovascular-healthy patients, based on the enrollment of only 3T cMRI scans of psychiatric patients in otherwise good condition. Nevertheless, the aneurysm prevalence of the study cohort (2.3–6.5%) seems to be within the range as supposed by the literature [12,13,14,15]. In conclusion, the study results need further validation, especially based on broader cohort characteristics and on a broader qualification spectrum of initially reporting radiologists.

Nevertheless, the study methodology and the presented NNS of an AI-powered cMRI screening allow us to balance the benefit of additionally detecting a few aneurysms that should be treated or followed up with the high burden of additional follow-up imaging for inconclusive or minor findings. A high number of cMRIs had to be screened to uncover relevant findings, e.g., quantified by an NNS of 152 to additionally identify highly suspicious findings (scores 2/3) with estimated UIAT scores balanced or in favor of treatment. On the other hand, a NNS of 26 for indicating a FU-cMRI or an additional DSA (Figure 6d) implies an almost 4% increase in examination workload solely due to these FU examinations, likely higher if also considering cases with more than only one necessary FU. These data, as derived, finally provide a possible database for future cost-effectiveness analyses.

Regarding study-related limitations, there is still a limited sample size, particularly regarding the low disease prevalence, which aimed to be represented by consecutive patient inclusion. Furthermore, the non-pathology-enriched cohort may introduce another selection bias, as the data were derived from a 3T MRI scanner specifically used for psychiatric patients, who are typically younger and in better physical condition compared to other in-hospital patients, potentially contributing to above-average image quality. Furthermore, a selection of cMRI datasets was made (after AI analysis) for the reference reading according to reasonable criteria, which nevertheless limits our statistics and diagnostic metrics calculation. Also, the single-reader assessment of our reference reading does not allow for inter- or intra-reader analysis. Finally, the single-center design raises questions about the generalizability of results, as outlined above, regarding the specific criteria of the cohort and the experience level of involved neuroradiologists.

Further studies are warranted to extend the results to broader spectrums of underlying cohorts, e.g., differentially considering representative outpatient and hospitalized cohorts. This might enhance the data basis—as initiated by this study—for following cost-effectiveness analyses, which will also need to take country-specific considerations into account, e.g., AI-related reimbursement issues. Furthermore, algorithm development enabling the analysis of other sequences beyond TOF-MRAs might possibly improve overall performance and would also extend a possible screening to a higher number of cMRI examinations.

## Data Availability

Anonymized data-sharing possibilities can be evaluated upon request in coordination with our institutional data protection office.

## Abbreviations

The following abbreviations are used in this manuscript:

ACA	anterior cerebral artery
AchA	anterior choroidal artery
Acom	anterior communicating artery
AI	artificial intelligence
AI-positive	MRIs with AI detections
AI-negative	MRIs without AI detection
cMRI	cranial magnetic resonance imaging
DSA	digital subtraction angiography
FPR	false positive rate
FU MRI	follow-up magnetic resonance imaging
ICA	internal carotid artery
MCA	middle cerebral artery
NNS	number needed to screen
NPV	negative predictive value
PACS	picture archiving communications system
PCA	posterior cerebral artery
Pcom	posterior communicating artery
PICA	posterior cerebral artery
PPV	positive predictive value
RFS	reference standard
SAH	subarachnoid hemorrhage
SUCA	superior cerebellar artery
TOF-MRA	time-of-flight magnetic resonance angiography
yo	years old
